# The TRUTH confirmed: validation of an intraindividual comparison of gadobutrol and gadoteridol for imaging of glioblastoma using quantitative enhancement analysis

**DOI:** 10.1186/s41747-021-00240-2

**Published:** 2021-10-12

**Authors:** Matthew J. Kuhn, Julia W. Patriarche, Douglas Patriarche, Miles A. Kirchin, Massimo Bona, Gianpaolo Pirovano

**Affiliations:** 1 University of Illinois College of Medicine at Peoria, 221 NE Glen Oak Ave, Peoria, IL 61636 USA; 2 A.I. Analysis, Inc., 1425 Broadway #20-2656, Seattle, WA 98122 USA; 3 Global Medical & Regulatory Affairs, Bracco Imaging SpA, Via Caduti di Marcinelle, 13, 20134 Milan, Italy; 4 Global Medical & Regulatory Affairs, Bracco Diagnostics, Inc., 259 Prospect Plains Rd. Building H, Monroe Township, NJ 08831 USA

**Keywords:** Contrast media, Crossover studies, Gadobutrol, Gadoteridol, Glioblastoma

## Abstract

**Background:**

Previous intraindividual comparative studies evaluating gadobutrol and gadoteridol for contrast-enhanced magnetic resonance imaging (MRI) of brain tumours have relied on subjective image assessment, potentially leading to misleading conclusions. We used artificial intelligence algorithms to objectively compare the enhancement achieved with these contrast agents in glioblastoma patients.

**Methods:**

Twenty-seven patients from a prior study who received identical doses of 0.1 mmol/kg gadobutrol and gadoteridol (with appropriate washout in between) were evaluated. Quantitative enhancement (QE) maps of the normalised enhancement of voxels, derived from computations based on the comparison of contrast-enhanced T1-weighted images relative to the harmonised intensity on unenhanced T1-weighted images, were compared. Bland-Altman analysis, linear regression analysis and Pearson correlation coefficient (*r*) determination were performed to compare net QE and per-region of interest (per-ROI) average QE (net QE divided by the number of voxels).

**Results:**

No significant differences were observed for comparisons performed on net QE (mean difference -24.37 ± 620.8, *p* = 0.840, *r* = 0.989) or per-ROI average QE (0.0043 ± 0.0218, *p* = 0.313, *r* = 0.958). Bland-Altman analysis revealed better per-ROI average QE for gadoteridol-enhanced MRI in 19/27 (70.4%) patients although the mean difference (0.0043) was close to zero indicating high concordance and the absence of fixed bias.

**Conclusions:**

The enhancement of glioblastoma achieved with gadoteridol and gadobutrol at 0.1 mmol/kg bodyweight is similar indicating that these agents have similar contrast efficacy and can be used interchangeably, confirming the results of a prior double-blind, randomised, intraindividual, crossover study.

## Key points

- Objective image assessment using artificial intelligence algorithms revealed no significant differences between gadoteridol and gadobutrol in terms of contrast enhancement of glioblastoma.
- Differences in concentration and minimal differences in relaxivity between gadoteridol and gadobutrol did not impact enhancement and visualisation of glioblastoma.
- The artificial intelligence software utilised helped to overcome potential reader-dependent variations in subjective image measurement and interpretation.

## Background

Traditionally, studies comparing gadolinium-based contrast agents (GBCAs) for contrast-enhanced magnetic resonance imaging (MRI) have relied on subjective qualitative assessment of images and the subjective placement of discrete regions of interest (ROIs) for quantitative enhancement (QE) measurements. The design of the study (intraindividual compared to interindividual) as well as image randomisation and anonymisation may help to overcome many potential issues related to subjective image assessment; however, these strategies do not help to overcome the underlying limitations of the human visual system. Fundamentally, the human visual system is not designed to easily identify differences between images when compared side-by-side. Rather, the human visual system works in abstractions, and consequently, it suppresses our sensitivity to differences when images are displayed side-by-side. The possibility to overcome these limitations with technology has long been recognised [1–4].

When GBCA imaging performance is compared it is necessary to compare not only the contrast-enhanced images but also the unenhanced images acquired immediately prior to GBCA administration since all that is bright in a contrast-enhanced image is not necessarily enhancing (*e.g.*, blood or fat). Conversely, a region could be isointense or even hypointense compared, for instance, with normal appearing white matter and still be enhancing. Because these phenomena are very difficult to perceive visually when images are displayed side-by-side, computational difference-detecting technologies have been developed to augment human capabilities when assessing contrast enhancement [5].

The traditional quantitative approach to overcoming challenges of the human visual system requires the manual placement of ROIs followed by volume quantification. Although this approach has advantages, there is nevertheless still the possibility that subjective visual assessment and/or subjective variations in the placement of ROIs may affect the results obtained, potentially leading to incorrect and misleading conclusions. Perhaps more importantly, by resorting to subjective visual interpretation in conjunction with discrete manual volume quantification, such approaches fail to capture the nuanced nature of information available in modern medical images. The use of an advanced QE analysis (QEA) technique may allow more precise measurements of contrast enhancement, thereby permitting more accurate comparison of the enhancement achieved with different GBCAs. The potential benefits of quantitative difference analysis technology for identification, characterisation, and quantification of visually subtle differences between images have been described previously [6, 7]. Briefly, QEA technology utilises multiple forms of artificial intelligence to construct a QE map (QEM) for each acquisition in which imaging features unrelated to contrast enhancement are separated automatically from features that enhance following GBCA administration. The QEM is a three-dimensional volume showing voxel-by-voxel what is enhancing, where, and by how much. QEA technology permits comparison of contrast enhancement between two acquisitions on a voxel-wise basis, permitting quantitative comparison of the degree of enhancement within a region between acquisitions.

To date, all intraindividual crossover studies that have compared GBCAs for contrast-enhanced MRI of brain tumours have used traditional approaches to image assessment. Whereas these studies have unequivocally shown that the enhancement achieved with the high r1-relaxivity GBCA gadobenate dimeglumine (MultiHance, Bracco Imaging, Milan, Italy) is significantly higher than with all comparator GBCAs when administered at equivalent dose under identical conditions [8–14], less clear-cut findings have been reported for comparisons amongst GBCAs that have roughly similar “standard” r1-relaxivity. Thus, whereas some authors report significantly better enhancement and imaging performance with gadobutrol (Gadovist/Gadavist, Bayer Healthcare, Berlin, Germany) compared to comparator agents [15–17] others, comparing the same GBCAs under identical conditions, report similar or non-inferior imaging performance for the comparator GBCA compared to gadobutrol [18, 19]. Studies reporting better imaging performance with gadobutrol [15–17] invariably ascribe the stated benefits to the twofold higher concentration of the gadobutrol formulation and to higher r1-relaxivity of the gadobutrol molecule [20–23].

Gadoteridol (ProHance, Bracco Imaging, Milan, Italy) is a macrocyclic GBCA that differs from gadobutrol only in that a hydroxypropyl group on the gadoteridol molecule is replaced by a trihydroxybutyl group on the gadobutrol molecule [18]. Despite this relatively minor variation in molecular structure, two intraindividual crossover studies [16, 17] have concluded that gadobutrol possesses superior contrast enhancement characteristics for brain tumour imaging when compared to gadoteridol at the same dose while only one study [18] has reported no differences between these two GBCAs. Notably, however, the current prescribing information for gadobutrol based on the results of key pivotal phase 3 central nervous system studies as reviewed by the United States Food and Drug Administration (FDA) states that “performances of Gadavist and gadoteridol for visualisation parameters were similar” [24].

We aimed to compare, in a nonsubjective, explorative manner using QEA software, the enhancement achieved with gadobutrol and gadoteridol in patients with histologically confirmed glioblastoma to validate, or otherwise, results obtained previously using subjective image assessment [18].

## Methods

This was a prospective assessment of MRI datasets from a subset of patients with histologically confirmed glioblastoma enroled into a prior multicentre, multinational, Health Insurance Portability and Accountability Act-compliant study that compared gadobutrol and gadoteridol at equimolar 0.1 mmol/kg bodyweight doses using a rigorous double-blind, randomised, intraindividual, crossover design [18]. Ethics Committee approval and informed patient consent was provided for the original study, as described previously [18]. The decision to prospectively re-evaluate a subset of patients from a prior study rather than enrol a new patient cohort in part reflected the need to directly compare our findings obtained using QEA techniques with findings obtained using traditional techniques in the same patient population, and in part ethical considerations over the administration of two GBCA doses given recent concern over potential risks associated with Gd retention, even though no signs or symptoms associated with retained Gd in the brain have yet been identified [25–28].

### MRI

Full details of the imaging protocol and findings of the original multicentre study are provided elsewhere [18]. All patients underwent 1.5-T brain MRI, receiving gadobutrol and gadoteridol at equivalent 0.1 mmol/kg bodyweight doses in two, otherwise identical MRI examinations, separated by at least 48 h to avoid carryover effects but in all cases less than 12 days (4 days or fewer in 15/27 patients, 5 to 10 days in 9/27 patients) to minimise the chance of measurable lesion evolution. The two examinations in each patient were performed using identical sequences comprising T1-weighted spin-echo (SE), T2-weighted fast spin-echo, and T2-weighted FLAIR acquisitions before contrast injection and T1 SE and three-dimensional T1-weighted high-resolution gradient recalled-echo acquisitions after contrast injection. Sequence parameters varied within predefined ranges necessitated by the use of different imaging systems at different centres [18]. However, the same MRI scanner, imaging planes, section prescriptions, and sequence parameters were used for both examinations in each patient. T1-weighted SE sequences were utilised in this study to compare imaging performance. The sequence parameters were as follows: repetition time 333–767 ms, echo time 7.7–16 ms, excitations 1–3, section thickness 4–5 mm, investigated volume 17 × 22–28 × 28 cm [18]. Contrast administration was performed at a dose of 0.1 mmol/kg in a randomised manner according to a prospectively scheduled patient randomisation scheme [18].

### Image evaluation using dedicated artificial intelligence software

All patients with histologically confirmed glioblastoma from the original intraindividual crossover study [18] who underwent both MRI examinations and had full-image datasets available were included in this new analysis that used dedicated artificial intelligence software. Images for this new analysis were evaluated in matched study pairs and in fully blinded fashion by an experienced neuroradiologist (M.J.K.; 33 years of experience) and a software engineer (D.P.; 8 years of experience) in consensus using dedicated artificial intelligence software (“Change Detector”; A.I. Analysis, Inc.) [6, 7].

**Fig. 1:**
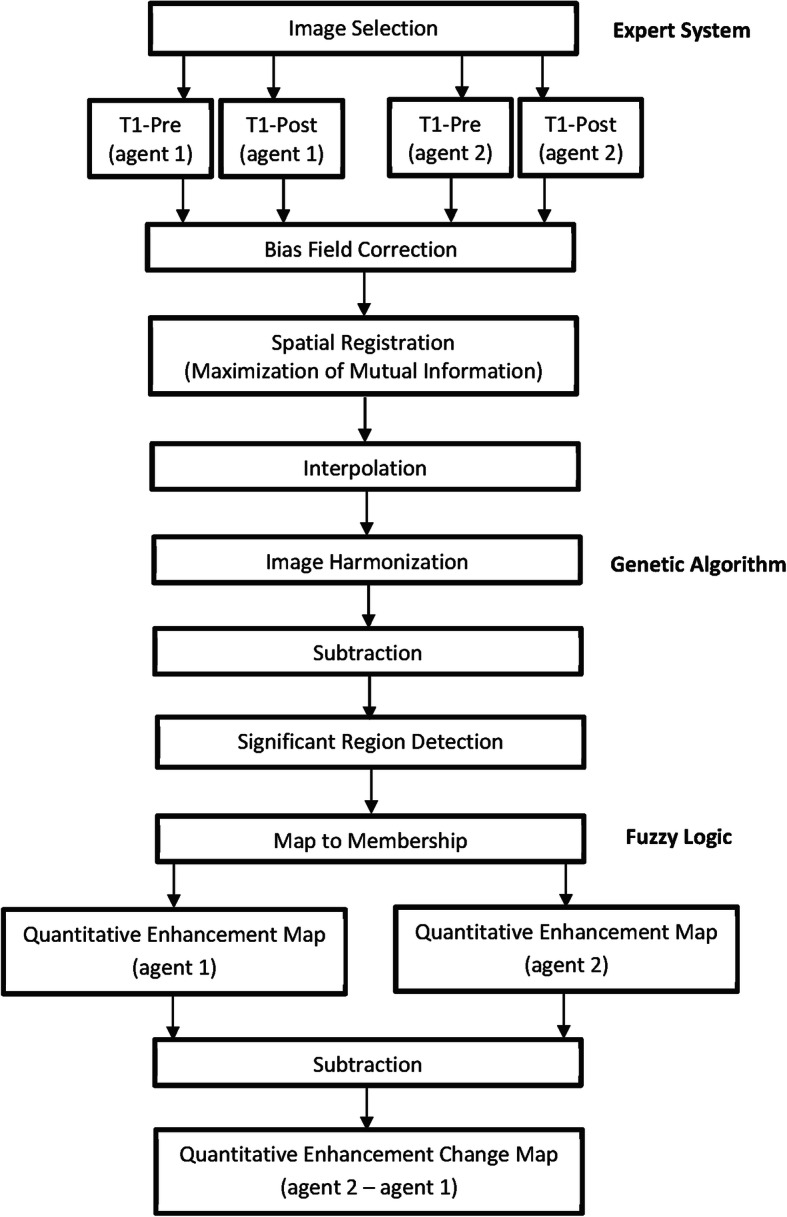
The quantitative enhancement (QE) analysis pipeline for comparing two GBCAs. T1-weighted unenhanced/T1-weighted contrast-enhanced pairs are acquired for both contrast agents. Bias field correction using the N4 algorithm is performed on all images, after which images are registered using an “all to one” approach. Subsequently, machine learning based intensity harmonisation is performed “all to one”. QE maps are calculated for each GBCA, and the difference between the QE map for the two GBCAs is calculated

At variance with traditional quantitative analysis which relies on volumetric assessment of voxels in a region of interest (ROI), *i.e.*, by simply calculating the number of voxels in the user-defined ROI, the QEA system used in this study utilises multiple forms of artificial intelligence (expert systems, fuzzy logic)/machine learning (genetic algorithms) in concert to construct QEM in which imaging features unrelated to the actual phenomenon of enhancement are separated from features that indicate enhancement. The QEA system makes calculations of enhancement in nuanced terms, rating the degree of enhancement within each voxel automatically and reproducibly on a fuzzy membership scale of 0.0 (nonenhancing) to 1.0 (maximally enhancing), such that the per-ROI sum of enhancement values provides not only an expression of the spatial extent of an enhancing region but also the degree of enhancement within that region. The software performs a pipeline of processing steps on the pre- and contrast-enhanced T1-weighted images from each paired data set corresponding to each patient (Fig. 1). These steps include the following:

- Bias field correction using the standard bias field correction algorithm N4
- Rigid registration using a ‘maximization of mutual information’-based approach
- Interpolation, so that all images for a patient are spatially aligned, with the same resolution
- Harmonisation of the intensity spectrums of the images using a proprietary approach, based on genetic algorithms
- Subtraction of each harmonised unenhanced T1-weighted image from its corresponding contrast-enhanced image, resulting in voxel-by-voxel enhancement values, followed by fuzzification of the voxel values (mapping of the enhancement value to the range 0.0 for no enhancement to 1.0 for maximal enhancement) resulting in a QEM for each contrast agent
- Subtraction of the QEM for one agent from that of the other to produce a map of the differences in enhancement between the two contrast agents

**Fig. 2:**
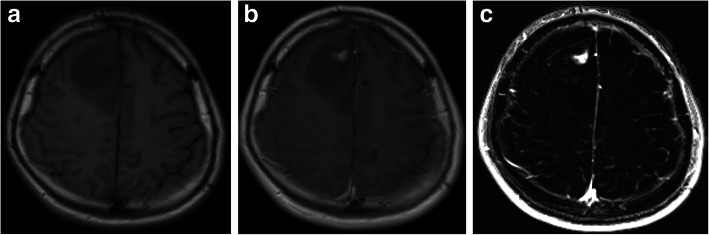
Paired and harmonised unenhanced (**a**) and contrast-enhanced (**b**) T1-weighted images are utilised to construct the quantitative enhancement (QE) volume (**c**) with enhancement scores from 0.0 (voxel is nonenhancing) to 1.0 (voxel is maximally enhancing). Partially enhancing voxels are ascribed a score between 0.0 and 1.0

**Fig. 3:**
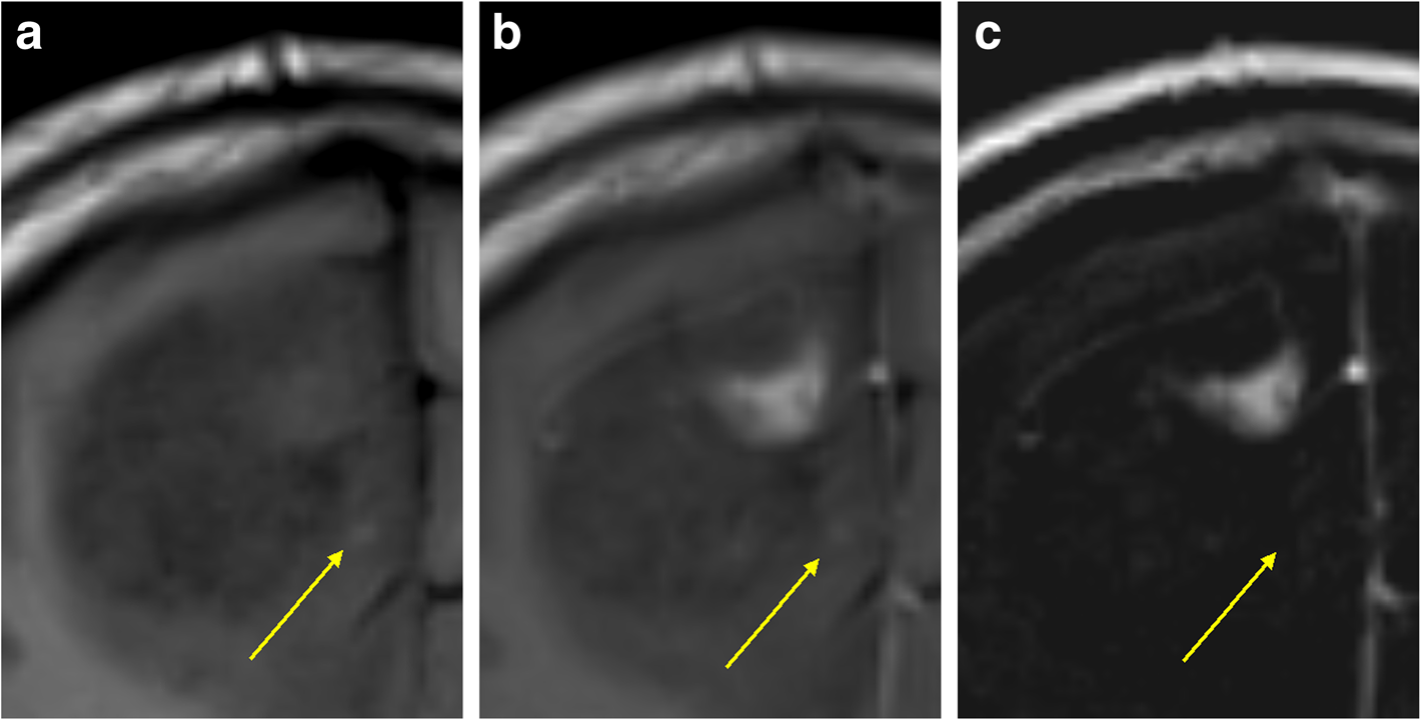
The quantitative enhancement (QE) analysis system considers both unenhanced (**a**) and contrast-enhanced (**b**) T1-weighted images when calculating the QE map (**c**) and thus knows that regions that are bright on the contrast-enhanced image (arrow) are not enhancing if they are equivalently bright in the unenhanced image

**Fig. 4:**
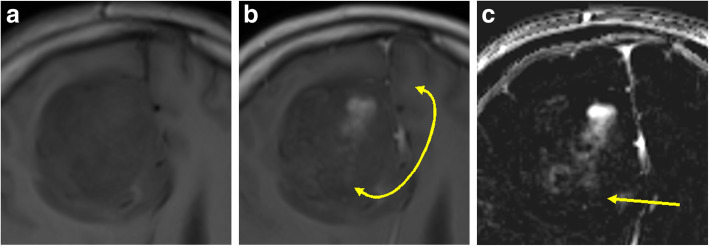
The quantitative enhancement (QE) analysis system analyses both unenhanced (**a**) and contrast-enhanced (**b**) T1-weighted images allowing accurate quantification of enhancement in regions that are much darker than normal appearing white matter (arrow on the QE map in **c**)

Initially, all four images for each case (unenhanced and contrast-enhanced T1-weighted SE images from both examinations) are registered by means of maximisation of mutual information. The users then place the ROIs manually around the lesion of interest. Each ROI could include just a single lesion or multiple lesions. By using linked cursors and flicker technology, the users see the location of the mouse pointer and ROI on all four images throughout the definition process and can define the ROI accordingly. Thereafter, all subsequent steps are fully automated. The QEA system constructs a QEM volume (Fig. 2c) from the paired unenhanced (Fig. 2a) and contrast-enhanced (Fig. 2b) images. Regions that are not enhancing are automatically assigned a QEM value of 0.0, while regions that are maximally enhancing are assigned a QEM value of 1.0. Levels of enhancement falling between nonenhancing and maximally enhancing are assigned QEM values between 0.0 and 1.0. By constructing a QEM based on comparison of the unenhanced and contrast-enhanced T1-weighted images, the system compensates for the underlying T1 intensity of the voxel and can detect and quantify enhancement that is imperceptible by visual review of the contrast-enhanced image. Because the QEA system considers both unenhanced and contrast-enhanced images when calculating the QEM, the system knows that regions that are bright in the contrast-enhanced image are not enhancing if they are equivalently bright in the unenhanced image (Fig. 3). By taking into consideration both unenhanced and contrast-enhanced images when calculating the QEM, the QEA system can correctly quantify enhancement in regions that are much darker than normal appearing white matter, by the fact that they are brighter in harmonised intensity in the contrast-enhanced image than in the unenhanced image (Fig. 4). For example, a region of a contrast-enhanced T1 image could be hypointense compared with normal appearing white matter but could be very slightly enhancing if it is brighter in terms of harmonised intensity than the corresponding region of the unenhanced T1 image. The registration and intensity harmonisation process permit construction of the QEM even when the unenhanced and contrast-enhanced images are acquired using different pulse sequences, resolutions, acquisition parameters, etc.

**Fig. 5:**
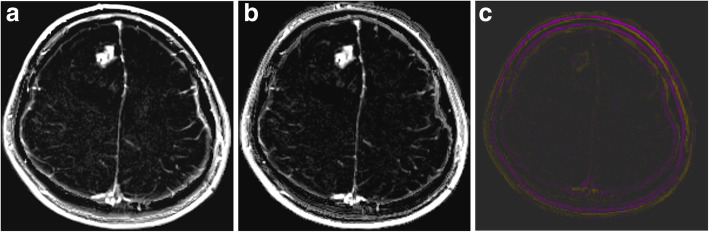
Comparison of quantitative enhancement (QE) maps for gadobutrol (**a**) and gadoteridol (**b**) reveals even very minor differences in contrast enhancement. In this case, the QE change map (**c**) shows that enhancement of the lesion rim (arrow) is slightly different between the agents. Purple indicates that enhancement is brighter after gadobutrol while yellow indicates that enhancement is brighter after gadoteridol

The QEA system permits direct quantitative comparison of images acquired with two GBCAs by computing a “QE change map” (Fig. 5). This permits clear visualisation of regions that are slightly more or slightly less enhancing between the two QEMs. Scatter charts of contrast-enhanced *versus* unenhanced voxel intensities within user-defined ROIs are created for each image set permitting direct comparison of two different MRI examinations in terms of voxel enhancement. ROIs are defined using ROI tools (pen, brush, and fill) built into the software to manually define the total extent of each tumour lesion in all slices. The extent of a lesion is defined to include any white matter region that is visibly abnormal (either hyperintense or hypointense) on contrast-enhanced T1-weighted images, including regions internal to the tumour that might not be hyperintense or hypointense. Quantitative analysis is then performed based on all voxels within the defined ROI. Nonenhancing voxels will cluster around a long snake-like region crossing the scatter chart (which will be linear when acquisition parameters are identical, or when the images have been harmonised; and non-linear otherwise), while enhancing voxels will deviate from this. Using these scatter charts, the patterns of enhancement can be visually compared between the two GBCAs. An enhancement scatter chart (QEM 1 *versus* QEM 2) can also be displayed, to permit direct comparison of the enhancement values for the voxels within each ROI for the two GBCAs. Pairs of GBCAs that exhibit similar enhancement characteristics generate points clustered around a line.

**Fig. 6:**
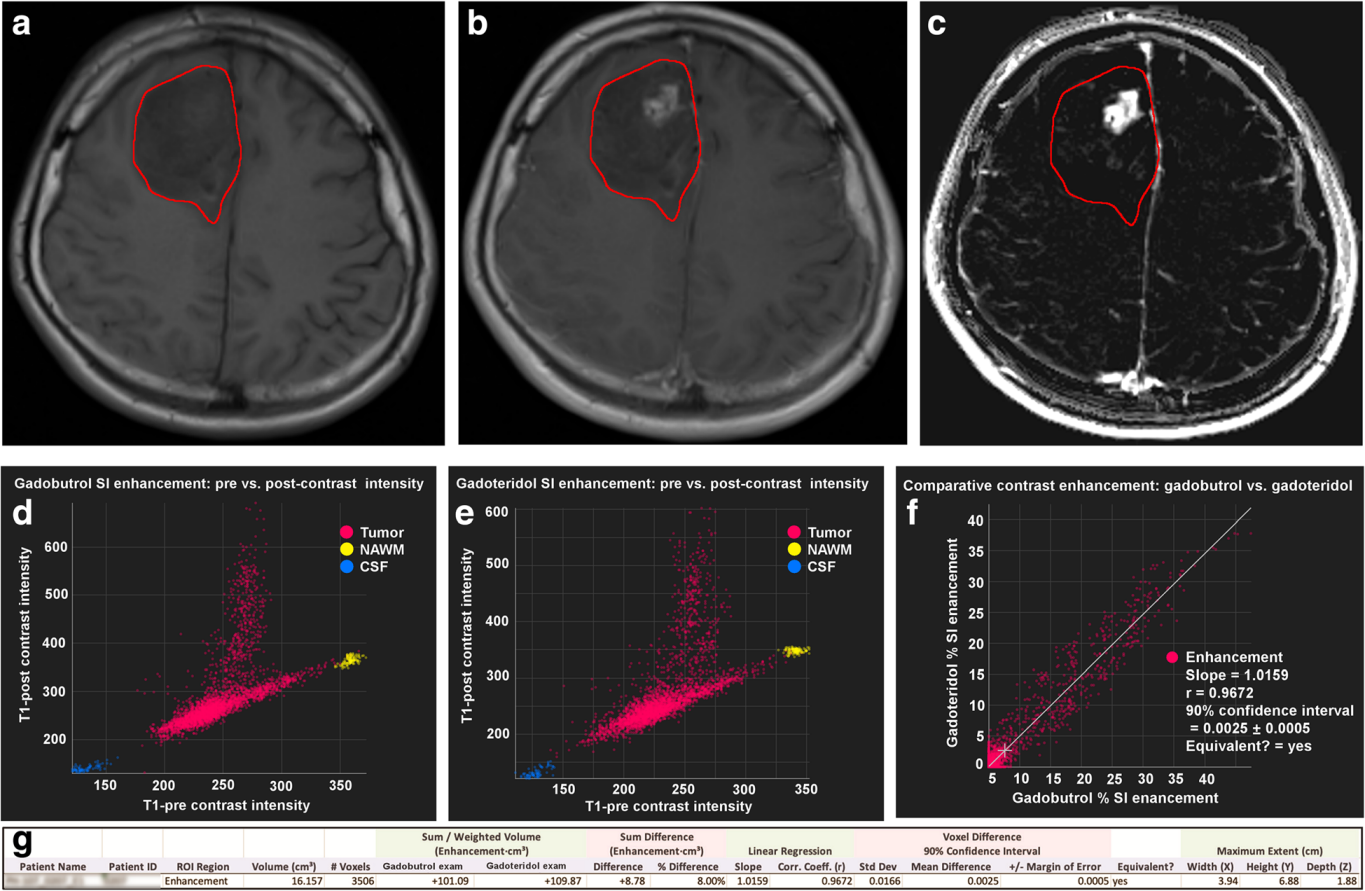
User interface and output of the quantitative enhancement (QE) analysis software for comparison of GBCA enhancement. A manually delineated region of interest (ROI) that includes the entire enhancing region for both GBCAs is placed on the spatially registered T1-weighted unenhanced (**a**) and contrast-enhanced (**b**) images; the system then automatically calculates the per-ROI sum of QE values for each GBCA (QE map is shown in **c**). ROI scatter charts (**d**, **e**) of voxel enhancement on T1-weighted contrast-enhanced *versus* T1-weighted unenhanced images reveals the patterns of enhancement for each of the two GBCAs. A scatter chart of the voxel-by-voxel QE value of GBCA 1 *versus* GBCA 2 (**f**) reveals differences, if any, between the enhancement achieved with each GBCA. Summary statistics (**g**) provide information on the volume, number of voxels, and maximum extent of the lesion as well as details of the QE map voxel enhancement obtained, represented as “Sum/Weighted Volume” for each GBCA and as the “Sum Difference” between GBCA 1 and GBCA 2. In this case, the volume of interest was 16.157 cm^3^ and 3506 voxels were measured. The per-ROI sum of QE values (enhancement × cm^3^) was + 101.09 for the first examination with gadobutrol and + 109.87 for the second examination with gadoteridol. The 90% confidence interval of the mean of the differences (0.0025) was within the equivalence zone indicating that the enhancement achieved can be considered equivalent for the two GBCAs

The volume, number of voxels, and maximum spatial extent within each defined ROI is automatically calculated. The system also computes the sum of the QEM values within each ROI, obtaining the per-ROI sum of QE values, which provides an expression of the overall degree of enhancement within the ROI, taking into consideration both spatial extent of enhancing portions, and degree of enhancement within each voxel. The difference between these per-ROI sum of QE values for the two corresponding agents is calculated. The slope and correlation coefficient of the QEM values for the two agents are computed. The difference between the per-ROI sum of QE values for the two GBCAs is calculated and a statistical equivalence test performed to determine whether the QEM values for voxels within the ROI are statistically equivalent for the two GBCAs or not. An “equivalence zone” for QEM values is defined as -0.2 to + 0.2; if the 90% confidence interval (CI) of the mean of the differences between the QEM values lies entirely within this “equivalence zone” the GBCA enhancement is considered equivalent for the two GBCAs (Fig. 6).

Note that the QEM may show real enhancement outside of the tumour region. For example, scalp, sinuses, blood vessels, and muscle also enhance after GBCA administration and there can be differences in this enhancement from one examination to the next. The “quantitative enhancement change map” will detect this. These differences can result for several reasons; for example, the patient may have gained or lost a bit of fat or have a little more or a little less inflammation in one exam relative to the other. This is the reason the user manually defines the ROI: to focus the analysis solely on the lesion.

### Statistical analysis

The number and percentage of patients were provided for categorical data. Summary statistics (mean, standard deviation, median, minimum, and maximum) were provided for continuous variables. The statistical tests were two-sided at the 0.05 level of significance with 95% confidence interval (CI).

Analysis was based on QEM intensity values obtained for voxels within each ROI on pre- and contrast-enhanced T1-weighted SE acquisitions for both GBCAs. Considerable differences in glioblastoma size across patients resulted in a large range of lesion volumes and, consequently, the number of voxels included in measurements (from 1,322 to 81,797 voxels). Therefore, in addition to the net QE value, a per-ROI average QE value for each lesion was calculated as the Net QE value divided by the number of voxels. Comparison of the Net QE value and the per-ROI average QE value of lesions after gadoteridol and gadobutrol administration was measured and compared using a paired *t*-test.

Bland-Altman analysis with 95% limits of agreement was performed to evaluate the agreement between the two contrast-enhanced MRI examinations in terms of QE. The 95% limits of agreement were defined as ± 2 standard deviations of the mean difference between the two examinations. In addition, scatterplots of the distribution of the two contrast-enhanced MRI examinations on assessments of both net and per-ROI average QE values were presented with linear regression fitted lines and Pearson correlation coefficients.

Finally, comparison of findings for differences in QE (gadoteridol *minus* gadobutrol) obtained using the current QEA system with findings for the three blinded readers in the original visual preference assessment [18] were compared using Spearman rank correlation analysis. The correlation was based on the difference in QE and the original score in the visual assessment where: 1 = gadoteridol better, 0 = equal, and -1 = gadobutrol better. Based on this analysis, a large positive correlation indicates a large difference in QE (gadoteridol better) and a greater preference for gadoteridol in the original visual assessment. Conversely, a negative correlation means the larger the difference in QE (gadoteridol better) the greater the preference for gadobutrol in the original visual assessment.

## Results

### Patients

Amongst 198 patients included in the original efficacy analysis population [18], 32 (16 males, 16 females; aged 55.9 ± 13.1 years (mean ± standard deviation); age range 19−73 years) had histologically confirmed glioblastoma and were eligible for this study. Of these 32 patients, 5 were excluded because of the absence of lesion signal enhancement on contrast-enhanced T1-weighted images on the generated QEM. The remaining 27 patients (14 males, 13 females; aged 56.5 ± 11.8 years; age range 30−73 years) were included in the analysis. These patients included 16 (8 males, 8 females) that received gadoteridol for the first examination and gadobutrol for the second, and 11 (6 males, 5 females) who received the two GBCAs in the reverse order. Histological confirmation of glioblastoma was determined from analysis of surgically excised tissue in 23 patients and biopsy sample in 4 patients.

### Net quantitative enhancement

**Fig. 7:**
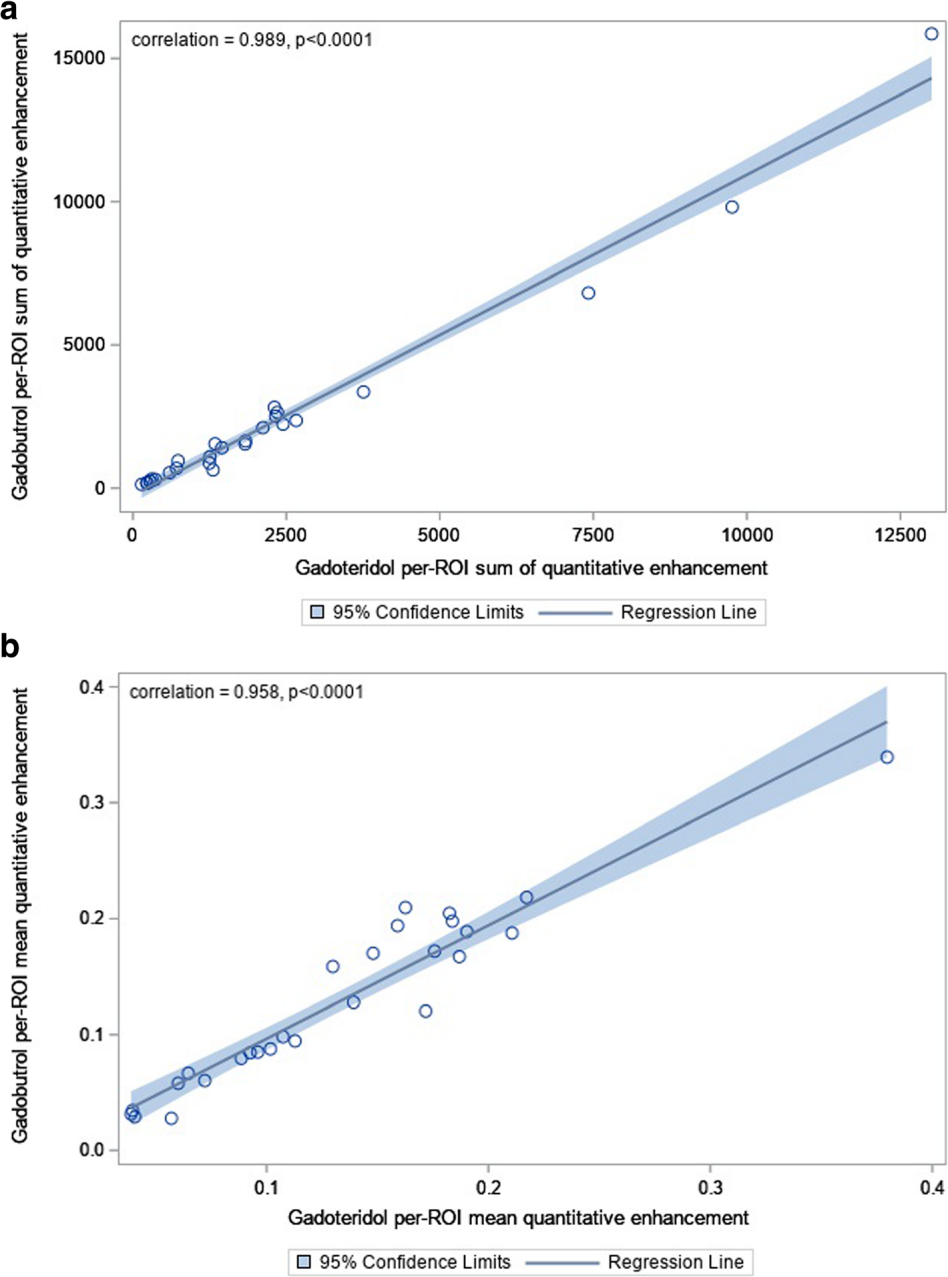
Per-region of interest sum of quantitative enhancement (**a**) and per-region of interest sum of mean quantitative enhancement (**b**) for gadoteridol *versus* gadobutrol

Values for net QE and per-ROI average QE for all 27 patients are shown in Table 1. No significant differences between gadoteridol and gadobutrol were observed when comparisons were performed of the net QE values (difference -24.37 ± 620.8, mean ± standard deviation, 95% CI -269.9, 221.2; *p* = 0.840) or the per-ROI average QE values (mean difference 0.0043 ± 0.0218, 95% CI − -0.00431, 0.0218; *p* = 0.313). Pearson correlation coefficients of 0.989 and 0.958 were obtained for determinations of net QE and per-ROI average QE, respectively (Fig. 7).

**Table 1:** Net quantitative enhancement and per-region of interest (per-ROI) average quantitative enhancement for 27 patients with histologically confirmed glioblastoma from the TRUTH study [18]

Patient	Net quantitative enhancement			Per-ROI average quantitative enhancement		
	Gadoteridol	Gadobutrol	Difference	Gadoteridol	Gadobutrol	Difference
1	2,660.6	2,355.5	305.1	0.096	0.085	0.011
2	225.3	196.1	29.1	0.040	0.034	0.005
3	7,421.5	6,814.4	607.1	0.139	0.128	0.011
4	1,829.6	1,533.0	296.6	0.113	0.095	0.018
5	736.3	949.3	-213.0	0.163	0.210	− 0.047
6	1,836.3	1,635.1	201.1	0.211	0.188	0.023
7	2,306.0	2,816.1	-510.1	0.130	0.159	− 0.029
8	711.3	695.5	15.8	0.176	0.172	0.004
9	2,445.9	2,225.5	220.4	0.093	0.084	0.008
10	3,755.9	3,356.3	399.6	0.380	0.339	0.040
11	9,759.5	9,814.0	-54.5	0.217	0.218	− 0.001
12	596.4	534.8	61.6	0.089	0.079	0.009
13	2,326.9	2,505.6	-178.7	0.184	0.198	− 0.014
14	308.1	317.2	-9.1	0.065	0.067	− 0.002
15	1,255.6	1,082.3	173.3	0.102	0.088	0.014
16	280.5	226.9	53.6	0.039	0.031	0.007
17	2,353.9	2,640.1	-286.2	0.182	0.205	− 0.022
18	277.3	248.1	29.2	0.187	0.167	0.020
19	364.5	304.8	59.8	0.072	0.060	0.012
20	13,009.7	1,5861.3	-2851.6	0.159	0.194	− 0.035
21	142.0	129.9	12.1	0.107	0.098	0.009
22	1,247.2	873.2	374.0	0.172	0.120	0.052
23	2119.5	2,103.0	16.6	0.190	0.189	0.001
24	230.1	164.5	65.6	0.041	0.029	0.012
25	1,339.9	1,539.6	-199.7	0.148	0.170	− 0.022
26	1,453.5	1,402.2	51.3	0.060	0.058	0.002
27	1,307.5	634.4	673.1	0.057	0.028	0.029
Mean ± SD	2,307.4 ± 3,045.4	2,331.8 ± 3,445.4	-24.4 ± 620.8	0.134 ± 0.074	0.129 ± 0.076	0.004 ± 0.022
*p*-value*			0.840			0.313

Positive difference values for individual patients indicate greater enhancement for gadoteridol; negative values indicate greater enhancement for gadobutrol. *p-value is from paired *t*-test for difference (gadoteridol *minus* gadobutrol)

**Fig. 8:**
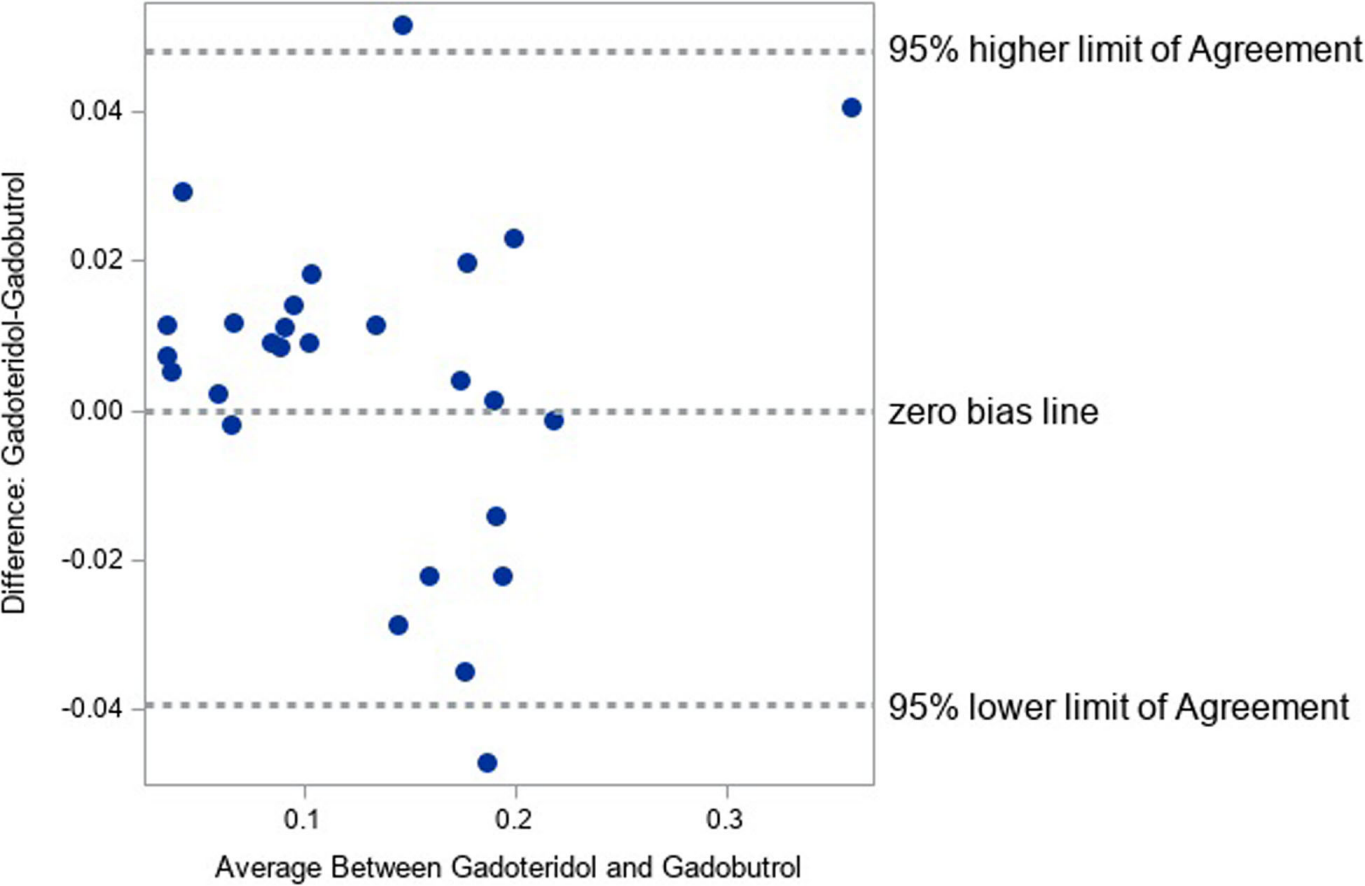
Bland-Altman plot for agreement of normalised net quantitative enhancement

**Fig. 9:**
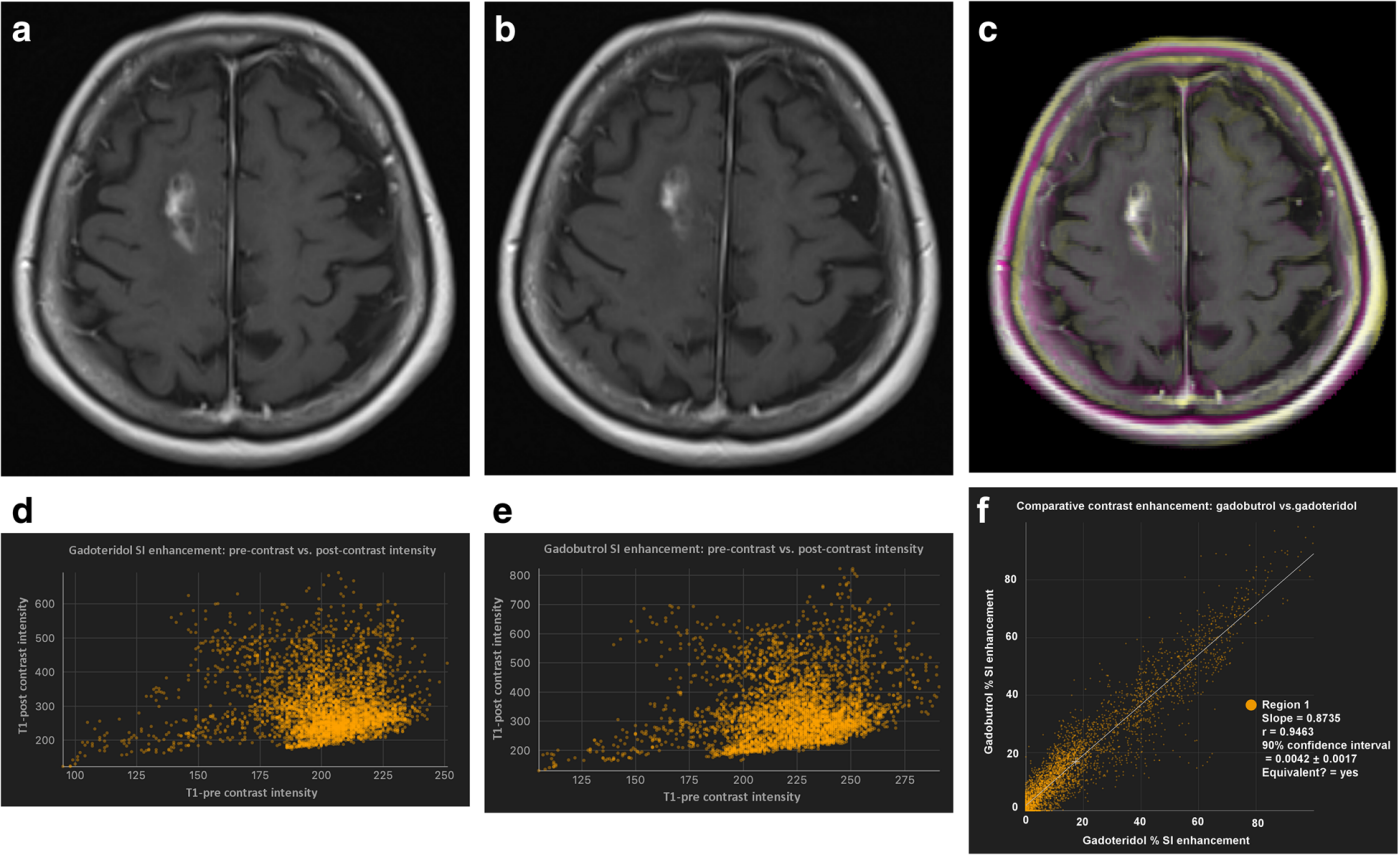
74-year-old woman with glioblastoma in the right centrum semiovale. Conventional T1-weighted SE contrast-enhanced images for the first exam with gadoteridol (**a**) and the second exam with gadobutrol (**b**) and the QE change map (**c**) where colour indicates regions differing in enhancement between the two exams. Scatter charts (T1-weighted contrast-enhanced *versus* unenhanced) of enhancing voxel regions of interest reveals the patterns of enhancement for gadoteridol (**d**) and gadobutrol (**e**). The net QE was +711.26 for gadoteridol (**a**, **d**) and +695.47 for gadobutrol (**b**, **e**). The gadoteridol *minus* gadobutrol difference (15.79, 2.22%) was not significant, and the 90% confidence interval for the mean of the differences fell entirely within the equivalence zone indicating equivalence for the two contrast agents, as demonstrated in the comparative scatter chart (**f**)

**Fig. 10:**
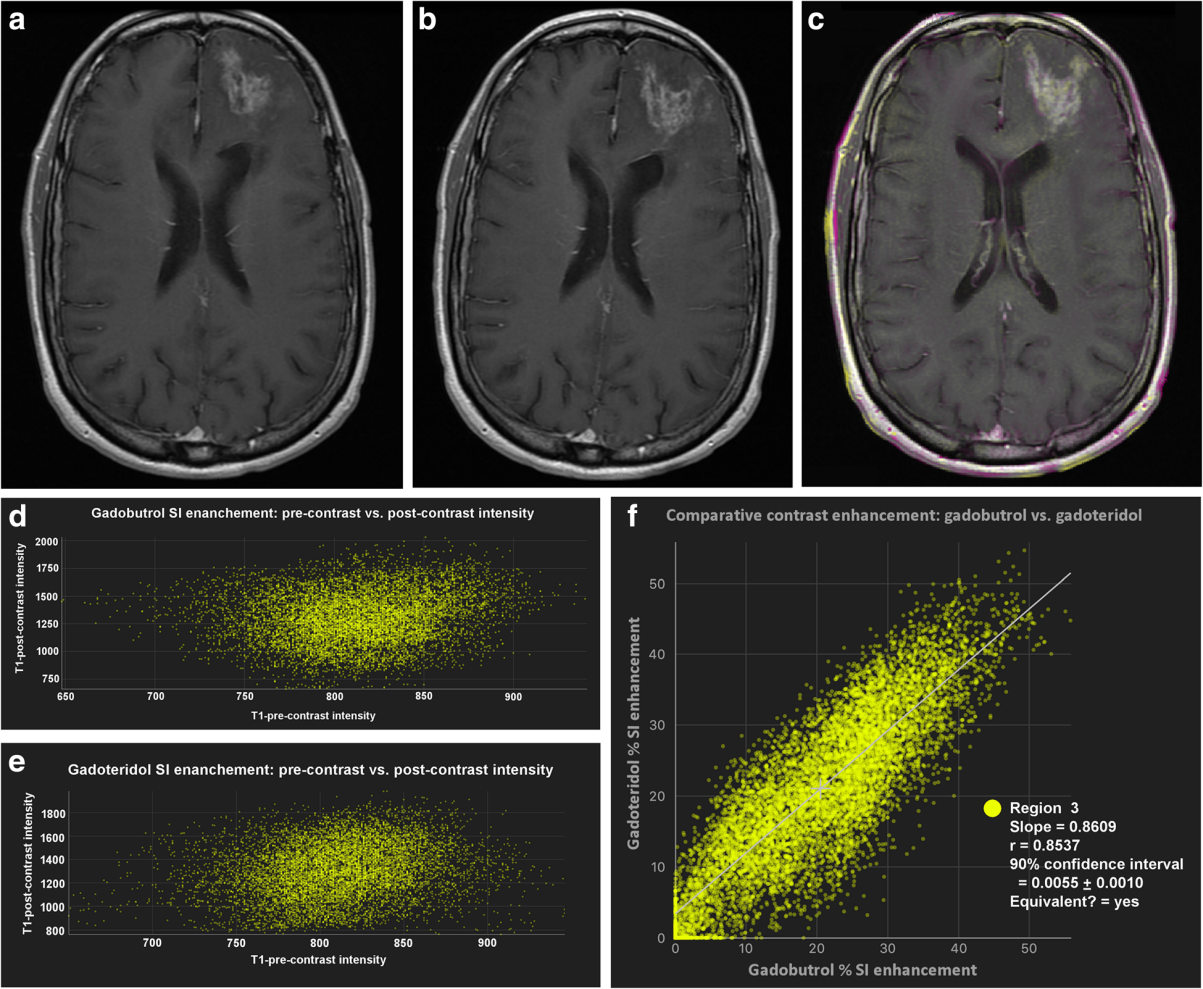
41-year-old man with glioblastoma in the left frontal lobe. Conventional T1-weighted spin-echo contrast-enhanced images for the first exam with gadobutrol (**a**) and the second exam with gadoteridol (**b**) and the QE change map (**c**) where colour indicates regions differing in enhancement between the two exams. Scatter charts (T1-weighted contrast-enhanced *versus* unenhanced) of enhancing voxel ROIs reveals the patterns of enhancement for gadobutrol (**d**) and gadoteridol (**e**). The volume of interest was 12.342 cm^3^ and 11,140 voxels were measured. The net QE was + 2,102.98 for gadobutrol and + 2,119.53 for gadoteridol. The gadoteridol *minus* gadobutrol difference (16.55, 0.79%) was not significant and the 90% confidence interval for the mean of the differences fell entirely within the equivalence zone (**f**), indicating equivalence for the two contrast agents

Notwithstanding the good correlation between measurements, better performance was obtained for gadoteridol-enhanced MRI in 19/27 (70.4%) patients, as demonstrated in Table 1 and shown by the Bland-Altman comparison of per-ROI average QE values in Fig. 8. However, the mean difference (0.0043) was close to zero, indicating the absence of any fixed bias between the two examinations. Likewise, the 95% limits of agreement between two examinations were very narrow (0.076), demonstrating high concordance between these two GBCAs. The comparable enhancement achieved with gadoteridol and gadobutrol, as highlighted by the roughly symmetric voxel enhancement along the mean line of the scatter chart and the correlation coefficients for the QEM values, is demonstrated in Figs. 9 and 10.

Spearman rank correlation analysis of findings obtained using the current QEA system with findings for the three blinded readers in the original visual assessment [18] revealed a significant correlation (*r* = 0.263; *p* = 0.017), confirming the accuracy and robustness of the QEA system.

## Discussion

Our study found no significant differences between gadoteridol and gadobutrol in terms of quantitative enhancement in patients with confirmed glioblastoma. Specifically, our objective analysis using dedicated QEA software found no significant differences between these two GBCAs when comparisons were made of net QE (mean difference -24.37 ± 620.8, *p* = 0.840) or per-ROI average QE (mean difference 0.0043 ± 0.0218, *p* = 0.313). Pearson correlation coefficients of 0.989 and 0.958 for determinations of net QE and per-ROI average QE, respectively, indicated near identical imaging performance for gadoteridol and gadobutrol for the enhancement of glioblastoma. This conclusion was strengthened by the results of Bland-Altman analysis which confirmed the absence of fixed bias between the two examinations.

In demonstrating similar imaging performance for gadoteridol- and gadobutrol-enhanced MRI, our findings confirm the results of the original TRUTH study [18] which compared these two GBCAs intraindividually in 229 patients with suspected or known brain tumours and found no differences in qualitative or quantitative enhancement based on subjective assessment by three fully blinded readers [18]. This was confirmed by the Spearman rank correlation analysis performed in our study which found significant correlation (*p* = 0.017) between our findings obtained using QEA software and those of the three blinded readers in the original visual assessment [18].

Conversely, the results of our study in patients with glioblastoma and those of the original TRUTH study in patients with both malignant and benign brain lesions [18] contrast with those of another large intraindividual crossover study performed in patients with both malignant and benign brain lesions which reported greater contrast enhancement and improved sensitivity and accuracy for the detection of malignant central nervous system disease with gadobutrol [17]. While this report should be viewed with caution as the results were presented as the averaged findings of three readers rather than as individual reader findings (as presented to the Food and Drug Administration for the USA approval of Gadavist [29]), they highlight the limitations and potential for bias when subjective image assessment alone is used to compare GBCA efficacy. The results of our objective analysis of a subset of patients with histologically confirmed glioblastoma from the TRUTH study [18] confirm the original subjective findings of the three blinded readers and validate the study conclusion that gadoteridol and gadobutrol at 0.1 mmol/kg bodyweight provide similar qualitative and quantitative imaging performance for the visualisation and diagnosis of these lesions.

The specific benefit of the dedicated algorithm used in this study is that it eliminates the inherent deficiencies of the human visual system to consistently identify differences between two similar images (for example, the contrast-enhanced images for GBCA 1 *versus* GBCA 2, the unenhanced *versus* contrast-enhanced images for each of GBCA 1 and GBCA 2, or the QEMs for GBCA 1 *versus* GBCA 2), when these images are displayed side-by-side. The need to repeatedly look back and forth to intentionally and explicitly compare each aspect of each subregion is both time-consuming and prone to error. The value of our quantitative and objective approach is that it accurately directs the viewer’s attention to the regions that are different between two images, thus making such differences highly conspicuous.

Our results confirm that any differences between gadoteridol and gadobutrol in terms of formulation concentration or r1-relaxivity have no clinical impact when imaging patients with glioblastoma. The ability of GBCAs to increase the signal intensity of the body tissues into which they distribute depends on the tissue concentration of the GBCA in question and on its specific r1 and r2 relaxivity values [14]. Given that the tissue GBCA concentration will be identical for equivalent GBCA doses (*i.e.*, 0.1 mmol/kg bodyweight in this case), any difference in signal enhancement on T1-weighted images will thus reflect differences in r1-relaxivity, assuming otherwise identical imaging conditions (*i.e.*, magnet field strength, sequence parameters, image acquisition timing, etc.). The similar imaging performances of gadoteridol and gadobutrol confirmed in our study indicate that the higher r1-relaxivity values reported for gadobutrol [20–23] are of insufficient magnitude to influence signal intensity enhancement. Numerous studies aimed at assessing the imaging performance of gadobutrol have referred to this GBCA as a “high relaxivity” agent [15, 17]. The results of our analysis confirm that gadobutrol does not deliver increased clinical utility as measured by clinically relevant increases in signal enhancement or lesion extent compared with gadoteridol and thus does not meet the criterion for a “high relaxivity” agent as defined by Kanal et al. [14].

The similar imaging performance of gadoteridol and gadobutrol at equivalent dose likely may extend to other MRI techniques such as dynamic or perfusion imaging. While studies comparing gadoteridol and gadobutrol at equivalent dose for perfusion MRI have still to be performed, a recent intraindividual comparison of gadobutrol and another half-molar GBCA, gadoterate meglumine (Dotarem, Guerbet, Aulnay-sous-Bois, France), at identical dose and delivery rate revealed no differences between these two GBCAs for dynamic contrast-enhanced T1-weighted perfusion MRI in patients with posttreatment glioma, leading the authors to conclude that these GBCAs are interchangeable in clinical routine [30]. Given the similar relaxivity values of all these GBCAs [20–23], it is to be expected that these agents would perform similarly across both dynamic and conventional contrast-enhanced delayed MRI techniques when injected at equivalent dose and delivery rate.

The choice of which GBCA to use in routine practice reflects not only an assessment of their respective risk-benefit ratios, but also consideration of various nonradiological factors such as price, availability, etc. Given the similar imaging performances of gadoteridol and gadobutrol and assuming similar commercial practicality, the choice of which GBCA to choose comes down to an evaluation of potential safety issues. Whereas prospective multicentre studies of gadobutrol and gadoteridol reveal no differences in terms of immediate contrast reactions [31–34], animal studies of gadolinium (Gd) retention suggest that gadoteridol is cleared much more rapidly from brain and other body tissues than gadobutrol [35–39] resulting in lower levels of retained Gd for longer periods of time. However, no signs or symptoms associated with brain Gd retention have yet emerged despite concerted research effort [25–28], and while the possibility of long-term effects on human health is an area of concern [40], no data are yet available that point to differences between the macrocyclic GBCAs in terms of impact on long-term human health.

A limitation of our study is that we included a relatively small subset of patients enroled into a previous double-blind, randomised, intraindividual, crossover study [18] rather than a prospective assessment of a new patient cohort. However, a benefit of our approach is that all data and information obtained using the new objective approach to image assessment could be directly compared with the findings from the conventional visual assessment approach, allowing corroboration of the previous conclusions or potentially revealing flaws in one or the other approaches. A second limitation is that the limited manual steps involved in the image analysis process were performed by only one expert neuroradiologist in combination with the software designer rather than by multiple neuroradiologists either in consensus or separately as in the original prospective study [18]. Although this approach prevented assessment of reproducibility and potential inter-reader variability, it highlights the potential value of the approach in terms of time- and work-saving opportunities. The high degree of reproducible automation in the QEA system used in this study would be expected to limit the potential impact of the user on the quantitative results that are generated, as noted elsewhere [41]. Finally, due to the explorative nature of the study, power calculation was not performed for this analysis.

In summary, our findings, obtained using dedicated QEA software, confirmed the results of a prior large-scale, multicentre, intraindividual crossover study in showing no significant differences between 0.1 mmol/kg bodyweight doses of gadoteridol and gadobutrol in patients with histologically confirmed glioblastoma. The QEA software utilised overcomes potential reader-dependent variations in subjective measurement and interpretation leading to stronger, more robust, and less misinterpretable conclusions.

## Data Availability

All data from the evaluation are included in the manuscript.

## Abbreviations

CI: Confidence interval
GBCA: Gadolinium-based contrast agent
MRI: Magnetic resonance imaging
QE: Quantitative enhancement
QEA: Quantitative enhancement analysis
QEM: Quantitative enhancement map
ROI: Region of interest
SE: Spin-echo

## References

[1] Simons DJ, Levin DT (1997). Change blindness. Trends Cogn Sci.

[2] Christianson S, Hofstetter HW (1972). Some historical notes on Carl Pulfrich. Am J Optom Arch Am Acad Optom.

[3] Patriarche J, Erickson B (2004). A review of the automated detection of change in serial imaging studies of the brain. J Digit Imaging.

[4] Radke RJ, Andra S, Al-Kofahi O, Roysam B (2005). Image change detection algorithms: a systematic survey. IEEE Trans Image Process.

[5] Curati WL, Williams EJ, Oatridge A, Hajnal JV, Saeed N, Bydder GM (1996). Use of subvoxel registration and subtraction to improve demonstration of contrast enhancement in MRI of the brain. Neuroradiology.

[6] Patriarche JW, Erickson BJ (2007). Part 1. Automated change detection and characterization in serial MR studies of brain-tumor patients. J Digit Imaging.

[7] Patriarche J, Erickson B (2007). Part 2. Automated change detection and characterization applied to serial MR of brain tumors may detect progression earlier than human experts. J Digit Imaging.

[8] Maravilla KR, Maldjian JA, Schmalfuss IM, Kuhn MJ, Bowen BC, Wippold FJ, Runge VM, Knopp MV, Kremer S, Wolansky LJ, Anzalone N, Essig M, Gustafsson L (2006). Contrast enhancement of central nervous system lesions: multicenter intraindividual crossover comparative study of two MR contrast agents. Radiology.

[9] Rumboldt Z, Rowley HA, Steinberg F, Maldjian JA, Ruscalleda J, Gustafsson L, Bastianello S (2009). Multicenter, double-blind, randomized, intra-individual crossover comparison of gadobenate dimeglumine and gadopentetate dimeglumine in MRI of brain tumors at 3 Tesla. J Magn Reson Imaging.

[10] Rowley HA, Scialfa G, Gao PY, Maldjian JA, Hassell D, Kuhn MJ, Wippold FJ, Gallucci M, Bowen BC, Schmalfuss IM, Ruscalleda J, Bastianello S, Colosimo C (2008). Contrast-enhanced MR imaging of brain lesions: a large-scale intraindividual crossover comparison of gadobenate dimeglumine versus gadodiamide. AJNR Am J Neuroradiol.

[11] Seidl Z, Vymazal J, Mechl M, Goyal M, Herman M, Colosimo C, Pasowicz M, Yeung R, Paraniak-Gieszczyk B, Yemen B, Anzalone N, Citterio A, Schneider G, Bastianello S, Ruscalleda J (2012). Does higher gadolinium concentration play a role in the morphologic assessment of brain tumors? Results of a multicenter intraindividual crossover comparison of gadobutrol versus gadobenate dimeglumine (the MERIT Study). AJNR Am J Neuroradiol.

[12] Vaneckova M, Herman M, Smith MP (2015). The benefits of high relaxivity for brain tumor imaging: results of a multicenter intraindividual crossover comparison of gadobenate dimeglumine with gadoterate meglumine (the BENEFIT study). AJNR Am J Neuroradiol.

[13] Kuhn MJ, Picozzi P, Maldjian JA, Schmalfuss IM, Maravilla KR, Bowen BC, Wippold FJ, Runge VM, Knopp MV, Wolansky LJ, Gustafsson L, Essig M, Anzalone N (2007). Evaluation of intraaxial enhancing brain tumors on magnetic resonance imaging: intraindividual crossover comparison of gadobenate dimeglumine and gadopentetate dimeglumine for visualization and assessment, and implications for surgical intervention. J Neurosurg.

[14] Kanal E, Maravilla K, Rowley HA (2014). Gadolinium contrast agents for CNS imaging: current concepts and clinical evidence. AJNR Am J Neuroradiol.

[15] Anzalone N, Scarabino T, Venturi C, Cristaudo C, Tartaro A, Scotti G, Zimatore D, Floris R, Carriero A, Longo M, Cirillo M, Cova MA, Gatti S, Voth M, Colosimo C (2013). Cerebral neoplastic enhancing lesions: multicenter, randomized, crossover intraindividual comparison between gadobutrol (1.0M) and gadoterate meglumine (0.5M) at 0.1mmolGd/kg body weight in a clinical setting. Eur J Radiol.

[16] Koenig M, Schulte-Altedorneburg G, Piontek M, Hentsch A, Spangenberg P, Schwenke C, Harders A, Heuser L (2013). Intra-individual, randomised comparison of the MRI contrast agents gadobutrol versus gadoteridol in patients with primary and secondary brain tumours, evaluated in a blinded read. Eur Radiol.

[17] Gutierrez JE, Rosenberg M, Seemann J, Breuer J, Haverstock D, Agris J, Balzer T, Anzalone N (2015). Safety and efficacy of gadobutrol for contrast-enhanced magnetic resonance imaging of the central nervous system: results from a multicenter, double-blind, randomized, comparator study. Magn Reson Insights.

[18] Maravilla KR, Smith MP, Vymazal J, Goyal M, Herman M, Baima JJ, Babbel R, Vaneckova M, Zizka J, Colosimo C, Urbanczyk-Zawadzka M, Mechl M, Bag AK, Bastianello S, Bueltmann E, Hirai T, Frattini T, Kirchin MA, Pirovano G (2015). Are there differences between macrocyclic gadolinium contrast agents for brain tumor imaging? Results of a multicenter intraindividual crossover comparison of gadobutrol with gadoteridol (the TRUTH study). AJNR Am J Neuroradiol.

[19] Maravilla KR, San-Juan D, Kim SJ, Elizondo-Riojas G, Fink JR, Escobar W, Bag A, Roberts DR, Hao J, Pitrou C, Tsiouris AJ, Herskovits E, Fiebach JB (2017). Comparison of gadoterate meglumine and gadobutrol in the MRI diagnosis of primary brain tumors: a double-blind randomized controlled intraindividual crossover study (the REMIND study). AJNR Am J Neuroradiol.

[20] Rohrer M, Bauer H, Mintorovitch J, Requardt M, Weinmann HJ (2005). Comparison of magnetic properties of MRI contrast media solutions at different magnetic field strengths. Invest Radiol.

[21] Noebauer-Huhmann IM, Szomolanyi P, Juras V, Kraff O, Ladd ME, Trattnig S (2010). Gadolinium-based magnetic resonance contrast agents at 7 Tesla: in vitro T1 relaxivities in human blood plasma. Invest Radiol.

[22] Shen Y, Goerner FL, Snyder C, Morelli JN, Hao D, Hu D, Li X, Runge VM (2015). T1 relaxivities of gadolinium-based magnetic resonance contrast agents in human whole blood at 1.5, 3, and 7 T. Invest Radiol.

[23] Szomolanyi P, Rohrer M, Frenzel T, Noebauer-Huhmann IM, Jost G, Endrikat J, Trattnig S, Pietsch H (2019). Comparison of the relaxivities of macrocyclic gadolinium-based contrast agents in human plasma at 1.5, 3, and 7 T, and blood at 3 T. Invest Radiol.

[24] Gadavist product label. https://www.accessdata.fda.gov/drugsatfda_docs/label/2011/201277s000lbl.pdf. Accessed 6 Oct 2020.

[25] Welk B, McArthur E, Morrow SA, MacDonald P, Hayward J, Leung A, Lum A (2016). Association between gadolinium contrast exposure and the risk of Parkinsonism. JAMA.

[26] Ackermans N, Taylor C, Tam R, Carruthers R, Kolind S, Kang H, Freedman MS, Li DKB, Traboulsee AL (2019) Effect of different doses of gadolinium contrast agent on clinical outcomes in MS. Mult Scler J Exp Transl Clin. 5(1):2055217318823796. 10.1177/2055217318823796. 10.1177/2055217318823796 PMC6378456 30800415

[27] Cocozza S, Pontillo G, Lanzillo R, Russo C, Petracca M, di Stasi M, Paolella C, Vola EA, Criscuolo C, Moccia M, Lamberti A, Monti S, Brescia Morra V, Elefante A, Palma G, Tedeschi E, Brunetti A (2019). MRI features suggestive of gadolinium retention do not correlate with Expanded Disability Status Scale worsening in multiple sclerosis. Neuroradiology.

[28] Vymazal J, Krámská L, Brožová H, Růžička E, Rulseh AM (2020). Does serial administration of gadolinium-based contrast agents affect patient neurological and neuropsychological status? Fourteen-year follow-up of patients receiving more than fifty contrast administrations. J Magn Reson Imaging.

[29] Available at https://www.accessdata.fda.gov/drugsatfda_docs/nda/2011/201277Orig1s000StatR.pdf Accessed 6 Oct 2020.

[30] Park J, Kim H, Shim W (2020). Comparison of dynamic contrast-enhancement parameters between gadobutrol and gadoterate meglumine in posttreatment glioma: a prospective intraindividual study. AJNR Am J Neuroradiol.

[31] Cho SB, Lee AL, Chang HW, Kim KA, Yoo WJ, Yeom JA, Rho MH, Kim SJ, Lim YJ, Han M (2020). Prospective multicenter study of the safety of gadoteridol in 6163 patients. J Magn Reson Imaging.

[32] Glutig K, Hahn G, Kuvvetli P, Endrikat J (2019). Safety of gadobutrol: results of a non-interventional study of 3710 patients, including 404 children. Acta Radiol.

[33] Prince MR, Lee HG, Lee CH (2017). Safety of gadobutrol in over 23,000 patients: the GARDIAN study, a global multicentre, prospective, non-interventional study. Eur Radiol.

[34] Tsushima Y, Awai K, Shinoda G, Miyoshi H, Chosa M, Sunaya T, Endrikat J (2018). Post-marketing surveillance of gadobutrol for contrast-enhanced magnetic resonance imaging in Japan. Jpn J Radiol.

[35] McDonald RJ, McDonald JS, Dai D (2017). Comparison of gadolinium concentrations within multiple rat organs after intravenous administration of linear versus macrocyclic gadolinium chelates. Radiology.

[36] Bussi S, Coppo A, Botteron C, Fraimbault V, Fanizzi A, de Laurentiis E, Colombo Serra S, Kirchin MA, Tedoldi F, Maisano F (2018). Differences in gadolinium retention after repeated injections of macrocyclic MR contrast agents to rats. J Magn Reson Imaging.

[37] Jost G, Frenzel T, Boyken J, Lohrke J, Nischwitz V, Pietsch H (2019). Long-term excretion of gadolinium-based contrast agents: linear versus macrocyclic agents in an experimental rat model. Radiology.

[38] Bussi S, Coppo A, Celeste R, Fanizzi A, Fringuello Mingo A, Ferraris A, Botteron C, Kirchin MA, Tedoldi F, Maisano F (2020). Macrocyclic MR contrast agents: evaluation of multiple-organ gadolinium retention in healthy rats. Insights Imaging.

[39] Bussi S, Coppo A, Bonafè R et al (2021) Gadolinium clearance in the first 5 weeks after repeated intravenous administration of gadoteridol, gadoterate meglumine, and gadobutrol to rats. J Magn Reson Imaging. 10.1002/jmri.27693 Online ahead of print 10.1002/jmri.27693 PMC8597020 33973290

[40] Available at: https://www.ema.europa.eu/documents/referral/gadolinium-article-31-referral-emas-final-opinion-confirms-restrictions-use-linear-gadolinium-agents_en.pdf. Accessed 6 Oct 2018

[41] Erickson BJ, Wood CP, Kaufmann TJ, Patriarche JW, Mandrekar J (2011). Optimal presentation modes for detecting brain tumor progression. AJNR Am J Neuroradiol.

